# Understanding the United States and Brazil’s response to obesity: institutional conversion, policy reform, and the lessons learned

**DOI:** 10.1186/s12992-015-0107-y

**Published:** 2015-06-10

**Authors:** Eduardo J. Gómez

**Affiliations:** International Development Institute, King’s College London, Chesham Building, Strand, WC2R 2LS UK

**Keywords:** Obesity, Institutions, Policy, United States, Brazil

## Abstract

**Background:**

In the United States (US) and Brazil, obesity has emerged as a health epidemic. This article is driven by the following research questions: how did the US and Brazil’s federal institutions respond to obesity? And how did these responses affect policy implementation? The aim of this article is therefore to conduct a comparative case study analysis of how these nations’ institutions responded in order to determine the key lessons learned.

**Methods:**

This study uses primary and secondary qualitative data to substantiate causal arguments and factual claims.

**Results:**

Brazil shows that converting preexisting federal agencies working in primary healthcare to emphasize the provision of obesity prevention services can facilitate policy implementation, especially in rural areas. Brazil also reveals the importance of targeting federal grant support to the highest obesity prevalence areas and imposing grant conditionalities, while illustrating how the incorporation of social health movements into the bureaucracy facilitates the early adoption of nutrition and obesity policies. None of these reforms were pursued in the US.

**Conclusions:**

Brazil’s government has engaged in innovative institutional conversion processes aiding its ability to sustain its centralized influence when implementing obesity policy. The US government’s adoption of Brazil’s institutional innovations may help to strengthen its policy response.

## Introduction

Obesity has emerged as a health epidemic in the United States (US) and Brazil. In the US, according to NHANES (National Health and Nutrition Examination Surveys) estimates, the number of obese Americans increased by 22 % from 1988 to 1994 [1]. By 2002, approximately 30 % of the population was obese, while in 2012 the number of adult obese was 34.9 % and 16.9 % for children [2]. This rise in obesity is mainly due to the increased consumption of high-caloric foods and sedentary life styles [1]. In Brazil, the percentage of overweight people increased from 43 % in 2006 to 53 % in 2014 [3], while the percentage of obese increased from 11.9 % to 17.9 % for the same period [3]. The introduction of cheap high-caloric foods, increased prices for nutritious foods, less physical activity and stress are attributed to obesity’s rise [4].

In light of these similar challenges, this article is driven by the following research questions: how did the US and Brazil’s federal institutions respond to obesity? And how did these responses affect policy implementation?

This article claims that the US and Brazil’s institutional responses were different. In the US, while the White House and US Department of Health & Human Resources (HHS) eventually introduced several prevention awareness campaigns, they nevertheless emphasized that state and municipal governments should take the lead in devising obesity prevention policies [5].^1^ However, in a context where most obesity cases in the US are found in distant, hard to reach rural areas [6], and where rural families often have difficulty accessing obesity prevention services [7–9], the federal government has not helped local governments by providing financial and human resource assistance, such as primary care physicians and healthcare workers effectively rendering obesity prevention services to these areas [10, 11]. In contrast, Brazil’s Ministry of Health not only introduced several prevention awareness programs, but it also effectively intervened in order to help municipal governments implement policy, providing funding and teams of primary care physicians, nurses, and healthcare workers through the Family Health Program (FHP, *Programa Saúde da Familia*). The FHP provides home visits and obesity prevention services to families in hard to reach rural areas and schools [12, 13]. Therefore, with respect to the types of institutions analyzed in this study, the main focus is on the reform of agency sub-divisions within federal health agencies. Both countries also differed in how they targeted the federal distribution of grant assistance and stipulations for grant funding for prevention programs, with Brazil providing grants to communities with the highest level of obesity while enforcing grant conditionalities onto the states [14, 15]; this has not been achieved in the US [16].

These differences in the US and Brazil’s response suggest important lessons to be learned. Indeed, the aim of this study is to conduct a comparative case study analysis of the US and Brazil in order to determine these lessons. Two key lessons emerge from this comparative analysis, drawn from the case of Brazil: first, the importance of converting preexisting federal primary healthcare agencies for achieving obesity prevention policy objectives; and second, the importance of social health movements within federal agencies shaping legislation based on sound nutrition and healthcare as a human right.

In seeking to explain the differences in institutional reform processes between the US and Brazil, the author applied and examined the institutional change literature discussing institutional *conversion* processes [17, 18]. Institutional conversion discusses the conditions under which institutions gradually change their organizational rules and procedures in order to achieve new policy objectives, without having to either reform or dismantle the existing institution [17, 18]. The application of conversion theory prompted the author to question if Brazil’s ability to convert preexisting health agencies followed a similar reform process and if not, what was unique about the case of Brazil and what new insights could be obtained.

### Context, policy evidence, and relevance

Brazil’s international recognition in the area of obesity prevention served as an intriguing and important comparison to be made with the US, considering the latter’s track record in being criticized for its lackluster policy response to obesity [19]. In 2010, at the International Congress on Obesity held in Stockholm, Sweden, organized by the *International Obesity TaskForce*, Brazil and the United Kingdom were selected as nations taking the lead in implementing effective obesity prevention policies [20]. In particular, the *TaskForce* highlighted Brazil’s success in regulating the food market, providing nutritious school meals through partnerships with agricultural producers, promoting healthy breastfeeding practices, monitoring obesity trends and its associated ailments through the Family Health Program (FHP) [20]. Other studies have argued that the Brazilian ministry of health’s increased investment in community-based obesity prevention programs, such as funding for parks, bike and running routes, and the provision of information to schools and local healthcare workers regarding the importance of physical activity and weight loss has provided “a model policy response” for other nations [21]. Public health researchers have also underscored the effectiveness of Brazil’s national dietary guidelines, namely the *Guia Alimentar Para a População Brasileira*, published in 2014 [22], which underscores the importance of consuming nutritional food to avoid malnourishment and obesity, community and family enjoyment in cooking meals [23]. Still others have praised Brazil’s ministry of health for its early efforts to work with the private sector in order to increase public awareness about nutritional content in foods [24]. Because of these accomplishments, scholars have argued that the US should adopt Brazil’s nutritional guidelines [23], while the director of the US Centers for Disease Control (CDC), Dr. Thomas R. Frieden, has stated that the CDC should learn from Brazil in the area of obesity prevention [25].

Recent evidence suggests that Brazil’s institutional and policy response to obesity has been effective. Specifically, scholars allude to the FHP’s effort to consistently visit households in order to provide obesity prevention services, such as information on good nutrition, exercise, and weight loss [12, 26]. This is important because in Brazil, many of the poor, especially in rural areas, often do not have the time (due to excessive work) or the finances needed travel for these preventative services. Because of this the FHP has been able to help families avoid the out-of-pocket expenses associated with traveling for services [12]. The FHP is also perceived as important because of the scarcity of nutrition experts at the community level, making FHP teams at times the only source of nutritional and wellness education available to families [27].

Other studies have shown that the FHP’s activities have had a direct impact on preventing obesity through weight loss. A quasi-experimental intervention in the Federal District of Brazil in 2009 revealed that the application of exercise and nutrition programs introduced through the FHP for a random sample population of overweight and obese individuals in the rural town of *São Sebastião* led to an increased reduction in weight loss and obesity [13]. Furthermore, some have argued that because of the FHP’s work with households, there has been a considerable reduction in hospitalizations for ailments associated with obesity, such as high blood pressure and diabetes [12, 26, 28–30]. Findings in this article also corroborate claims that the ministry of health’s funding of public parks and exercise programs in several cities has led to an increase in physical activity and the prevention of chronic illness [31, 32].

But on what basis is Brazil’s institutional response to obesity important in the US context? It is important because like Brazil, in the US obesity is highly prevalent in urban and hard to reach, distant rural areas, especially among low-income groups [33–35]. As in Brazil, moreover, the rate of obesity in the US is growing faster in these poor rural versus wealthier urban areas [33–35]. Similar again to Brazil, the poor in the US, especially in distant rural areas, often cannot seek medical attention due to distance, funding for travel, and time away from work [8, 36]. While community health centers do exist in the US, its staff do not make homes visits, while these centers are also hard to reach [8]. In this context, obese rural residents often lack adequate obesity prevention services, due to limited infrastructure and medical attention ([8, 33, 35, 37]); because of this, US health officials may stand to gain from adopting a federal assistance program similar to Brazil’s FHP.

## Methodology

This article adopted a comparative case study design and qualitative methodology to comparative health policy analysis. The purpose of the comparison between the US and Brazil was to highlight their unique historical, institutional, and policy contexts, as well as differences in causality and policy outcomes [38]. Following George and Bennett [38], this study points to the advantages of comparing a small number of cases in order to highlight the conditions under which particular causal mechanisms are present, their uniqueness, if they are present in other countries, and to what extent we can learn from them. With respect to the selection of cases, the author capitalized on the advantages of selection bias, i.e., selecting cases based on their known values on the dependent variable [38, 39]. By selecting the case of Brazil’s internationally recognized positive response to obesity versus, as mentioned earlier, the converse for the US, this provided an intriguing comparison yielding potential lessons for the US; moreover, this method was done in order to discover and explain causal processes not emphasized in the literature and therefore provide new insights into explaining the uniqueness of the Brazilian case. Finally, the US and Brazil were chosen for several similar reasons, such as the heightened increase in obesity cases in rural areas; the high degree of healthcare decentralization present, posing challenges to effective inter-governmental policy coordination and implementation; and finally, historically similar limitations in human resource and infrastructural capacity in poor rural areas [40]. These cases were also chosen because of the ample amount of published information on the topic, the author’s ability to read in Portuguese, and because of the author’s strong contextual knowledge of both nations, which facilitated the acquisition of data.

With respect to the qualitative data used in this study, the author used peer-reviewed journal articles, government documents, and reports published by think tanks, which were used to support the author’s causal and factual claims. Statistical data on government expenditures and epidemiological data were obtained from the departments of health in both countries for the same purpose.

## Obesity policy and institutional change theory

A brief review of the literature discussing the design of obesity prevention policies helps to situate the research in this article and to highlight differences between Brazil and the US, as well as Brazil’s innovative response. One area of research emphasizes the importance of policies addressing the broader social and economic environment contributing to obesity, such as the regulation of food systems, infrastructure, and policies promoting physical activity, which can be implemented at different levels of government [41]. For example, policies that lower prices for healthy foods, regulations ensuring the proper labeling of nutritional content, laws prohibiting the marketing and sale of fatty foods, and urban planning and infrastructure promoting increased physical activity, such as parks and walkways, have been implemented in several nations to prevent obesity [16, 42–46]. These policies focus on altering the social and economic environment in order to help individuals make healthy choices while providing opportunities to live healthier lifestyles [43, 45, 46].

Others have instead emphasized the important role of political leadership in prevention policy, such as education and advocacy [44, 46]. Lanigan [44] emphasizes the importance of politicians’ willingness to use the media to educate families about the importance of nutrition and exercise [44, 46]. As seen in the US with First Lady Michelle Obama, politicians may at times use the media to express their own personal family struggles with obesity in order to establish rapport and a connection with families, while encouraging them to seek preventative information through government obesity programs [40]. Alternatively, Swinburn [46] underscores the importance of governments engaging in advocacy campaigns for healthier family lifestyles, through a variety of social media and programmatic efforts, and the increased regulation of food content and marketing to children with the hopes of kindling greater social responsibility within the private sector.

Other obesity prevention policies instead focus on how institutions, such as governments, private employers, and schools create policies to influence individual behaviors, such as the choice to eat well and exercise. Good examples include the creation of wellness and nutritional campaigns and physical fitness activities within government agencies, businesses, and schools [45]. With respect to schools, these policies often entail federal and/or state government efforts to not only establish nutrition and physical education standards, but also staff efforts to reach out to families and to educate them, both through workshops and the provision of information, about the importance of proper nutrition and exercise [16, 43, 44].

As discussed shortly, Brazil and the US’ policy approach to obesity prevention comports with the aforementioned literature emphasizing the need to change the social and economic environment contributing to obesity, such as through increased federal funding for public parks and school gyms promoting physical fitness. However, in contrast to the US, Brazil’s policy approach critiques the literature emphasizing the importance of political leadership in obesity awareness and prevention, institutional approaches to shaping individual behavior through school outreach programs, and polices focused on providing primary care services.

Indeed, unlike the US, political leaders in Brazil have not displayed strong leadership in using the media to increase family awareness and interest in obesity prevention [47]. This in large part reflects presidential administrations’ overwhelming concern with tackling poverty, malnutrition, and hunger [47]. Instead, obesity prevention policy has been the product of bureaucratic interest and preexisting experiences stemming from a long policy history of government-sponsored nutritional campaigns and pro-active prevention in primary healthcare [48]. Moreover, the aforementioned institutional approach to providing obesity prevention policies shaping individual behavior is somewhat different in Brazil. For example, while schools certainly provide nutritional guidance and physical education to children [49], the ministry of health further supplements these efforts by meeting with families directly, through the FHP program, in order to further ensure that families obtain the nutrition and wellness information that they need. The case of Brazil therefore suggests that the policy literature focusing on institutional programs affecting individual behavior should consider how federal agencies *supplement* preexisting school outreach programs.

Nevertheless, Brazil’s obesity prevention policies also contribute alternative insights into what Sacks [45] refers to as the “downstream” approach to obesity prevention. According to Sacks [45], this approach emphasizes individuals’ usage of primary and secondary healthcare services for the provision of nutritional and wellness information. Here, what is important is that there is an ample supply of nutritionists and dieticians within hospitals that individuals can visit to obtain primary care services for information on nutrition, fitness, and the monitoring of bodily functions. In contrast to this approach, the FHP’s focus in Brazil has been not to supply hospitals but to increase the number of nutritionists and dieticians working *outside* of hospitals by visiting households and ensuring that those that cannot visit hospitals receive the preventative information that they need.

In addition to obesity policy, students of comparative health politics have also become interested in analyzing institutional change processes, which often entails the reform of the bureaucracy, representative political institutions, and their policy effects [17, 50, 51]. This article’s effort to explain the gradual adaptation of Brazil’s FHP and its implementation of obesity prevention policies resonates with a growing body of literature focused on gradual institutional change processes [17, 18, 50, 52]. More specifically, findings from the case of Brazil comports with this literature’s claim that endogenous institutional change can occur in the absence of exogenous shocks (e.g., economic crisis), and that much of the reform that takes place within institutions is gradual, often clandestine, shaped by reform actors^2^ employing different types of strategies to alter the purpose, design, and objectives of institutions [17, 18].

In this literature, scholars have highlighted several types of reform strategies that institutions pursue. A process of institutional *displacement* occurs when previously marginalized actors within institutions introduce alternative institutional designs replacing pre-existing ones and their policies [17, 18, 53]; this is achieved by gradually discrediting previous institutional designs and policies by revealing their repeated failures and dwindling support, while highlighting the effectiveness of the proposed alternative [17, 18, 53]. An instance of institutional *drift* explains how a change in the external environment, such as worsening economic conditions, weakens existing institutions and their policies, in turn providing an opportunity for alternative institutions to arise [17, 18, 50, 53, 54]. In this instance, the neglect to reform existing institutions in order to adapt to new environments leads to a gradual abandonment of the institution in support of another [17, 18, 50, 53, 54]. And in an instance of institutional *layering*, actors realize that they do not have the resources and influence needed to reform existing institutions on their own and, instead, seek to work around them by introducing formal amendments (such as to constitutions) and/or additional policies to existing institutions with the goal of achieving reformers’ alternative objectives [17, 18, 51, 53]; this process is facilitated by reform actors ensuring that those supporting prior institutional designs remain content while nevertheless crafting new ideas, amendments, policies, and coalitions to achieve reforms’ policy goals [17, 18, 51, 53].

However, in this article Brazil’s institutional innovations in response to obesity were more closely related to the literature emphasizing institutional *conversion* [17, 18, 50, 53]. Conversion theory was most applicable because Brazil’s Family Health Program (FHP) was not discredited for failing to respond to the obesity epidemic, thus being *displaced* with another institution. Nor did the FHP *drift* out of significance, as it was gradually transformed in adaptation to the epidemic. And an instance of *layering* did not occur because FHP actors did not create additional policy amendments, agency divisions, or policies going against its approach to obesity prevention.

A conversion process occurs when reform actors within institutions re-interpret, re-direct, and re-purpose the institution’s design, ideas, meaning, and policies for new policy objectives [17, 18, 50, 52, 53, 55]. For instance, here, actors re-purpose bureaucratic agencies for new policy objectives, gradually adapting to changes in the environment. For a conversion process to unfold, institutional rules must be ambiguous and contested among institutional actors [17, 50]. Reform actors must use this ambiguity to propose alternative institutional procedures and goals, while using their funding, influence, and coalitional allies to achieve their alternative policy objectives [17, 18, 50, 55].

A conversion process also occurred in Brazil. As discussed shortly, realizing the need to respond to the increased prevalence of obesity, by the early-2000s ministry of health officials began to assign new roles and responsibilities to FHP teams, going beyond their traditional roles of general primary healthcare. FHP teams were delegated the task of providing several types of obesity prevention services to households and schools.

Nevertheless, the case of Brazil revealed a limitation to the institutional conversion literature. This literature does not consider how and why reform actors within ministries of health, after engaging in a process of conversion, seek to introduce new agency subdivisions and policies that reinforce, strengthen, and therefore *consolidate* conversion processes. As the case of Brazil will illustrate, ministry of health officials may invest in additional human resources and create new agency sub-divisions in order to consolidate a conversion process, thus making institutions all the more durable, adaptable, and capable of achieving their policy objectives. Such was the case with the ministry of health’s subsequent creation of specialized obesity prevention teams, namely the *Núcleo de Apoio á Saúde da Família* (NASF), which were explicitly created to assist FHP workers in providing obesity prevention services.

## Public health policy implementation in the US and Brazil

While the US and Brazil are vast democracies with nationally elected Presidents (for 4 year terms, with the possibility of re-election) wielding strong executive powers (decree and veto authority), an elected Congress (House and Senate in the US, versus the Deputy of Chambers [House] and Senate in Brazil), and an open-list electoral system generating intra- and inter-political party competition over the representation of constituent interests [56], the implementation of public health policy faces fewer hurdles in Brazil. While public health agencies in both nations are autonomous in creating policies and regulations [57, 58], Brazil’s agencies appear to be more successful at securing congressional funding and political support for policy implementation. This is in large part the result of preexisting normative commitments to the provision of universal healthcare as a human right, a key component of the transition back to democracy in 1986 [59]; this context has facilitated the bureaucracy’s ability to obtain the Chamber of Deputies and Senate’s support for the ongoing financing of public health policies. In contrast, the Department of Health & Human Services in the US has at times in the past faced considerable difficulty obtaining financial support for new policies [60]; the absence of a shared normative commitment to healthcare as a human right [60], and conflicting partisan views over the government’s role in public health appear to have been the main culprits [61].

Finally, strong cooperative inter-governmental relationships and a long tradition of federal bureaucratic intervention in public health also appears to have facilitated Brazil’s ability to coordinate with the states for policy implementation. While both nations are large federations with over 20 states (50 in the US, 27 in Brazil) with the decentralization of public health policy responsibility, Brazil’s ministry of health has a much longer tradition of intervening and coordinating with the states to implement public health legislation [57]. In this context, governors and mayors have been more welcoming of federal assistance to implement policy. In contrast, by the early-20^th^ century the US Public Health Service (PHS) decentralized the implementation of public health policy to the states [62]. Consequently, when compared to Brazil, the PHS has far less experience intervening and working with the state governments, while governors have often preferred to implement public health legislation on their own [63].

## US response

### Political calls to action

In 1974, the Department of Health & Human Services (HHS) and the National Institutes of Health (NIH) began to disseminate reports about America’s obesity epidemic. However, the White House and the Congress were essentially unresponsive [5, 64]. While President Jimmy Carter (1977-1981) recognized the issue and expressed concern, no national policies were recommended [65]. The Congress was also unresponsive due to pressures from business and agricultural producers [64, 65]. The Bill Clinton administration (1992-2000) received perhaps the most criticisms from special interest groups, such as the *National Association for Fat Acceptance* (NAFA), the media, and the bureaucracy [40]. The White House and most of the Congress did not respond during this time because of their view that obesity was not a national health threat, for several reasons, ranging from the absence of obesity’s affects on the national security to the economy [40].

The government’s attention and response to obesity quickly changed under the George W. Bush (2000-2008) and Barack Obama administrations (2008-). Bush’s seemingly obsessive concern with physical fitness and health motivated him to elevate the discussion about obesity [66]. President Obama and First Lady Michelle Obama nevertheless seemed to be more concerned with escalating childhood obesity cases and their children’s health [40, 67].

Nevertheless, the state governments had already started to implement obesity policies. In the US’ decentralized context, considering their primary responsibility for creating and implementing public health legislation, the states were early policy innovators in this regard [5]. By 2004, for example, Arkansas’ Governor Michael Huckabee succeeded in implementing legislation mandating that all schools measure students’ BMI index, while providing “weight report cards” to families; several states, such as California, Florida, and Pennsylvania, adopted these policies [68]. These early state government efforts have led some scholars to claim that President Bush’s national efforts were inspired by these local innovations [5].

The Bush and Obama administrations’ personal interest in obesity motivated them to initiate federal campaigns and legislation. Under Bush, the Federal Obesity Prevention Act of 2008 was implemented [69]. This Act created a federally-coordinated effort to address the epidemic; it also led to the creation of a National Coordinator of Obesity Initiatives and required the US Department of Health & Human Services director to convene a taskforce undertaking the following: 1) establish a government-wide strategy for prevention and reduction of obesity; and 2) coordinate effective interagency action and prioritize cooperation among federal agencies [69]. This Act also required agency heads to conduct reviews about how their budgets impacted physical activity, nutrition, and obesity, while requiring the US Comptroller General to review all agency budgets in order to determine how program budgets impact physical activity, nutrition, and obesity in the states [69]. Additionally, the 110^th^ Congress (2007-2009) enacted a spate of obesity prevention initiatives, such as the *National Youth Sports Week*; the *National Physical Education and Sports Week*; the *Healthy Foods for Healthy Living Act*, which authorized the US Department of Agriculture to provide grants to the states to encourage the consumption of fruits and vegetables while requiring Medicare and Medicaid to cover services for obesity prevention/treatment; the *Menu Education and Labeling Act*, which required restaurants with 20 or more chains to post calorie counts and other nutritional information; and the *Improved Nutritional and Physical Activity Act*, which required the states to use preventative health service block grants for community initiatives designed to address obesity and eating disorders [70]. These initiatives were mainly designed to improve consumer nutritional choices and improve the environment in response to obesity [70].

The Obama administration entered office with more of a focus on combating childhood obesity. In February 2010, First Lady Obama created the *Let’s Move!* campaign. This initiative called for a coordinated effort from federal and state governments, the private sector, and families to work together in curbing the spread of childhood obesity. *Let’s Move!* pursued the following goals: 1) to help parents make healthy food choices; 2) to create healthier schools; 3) to increase physical activity; and 4) to increase access to healthy and affordable foods [67]. *Let’s Move!* was followed up by an executive order from President Obama titled *Task Force on Childhood Obesity*, which similarly called for a coordinated effort between the aforementioned stakeholders in order to: 1) empower parents and caregivers to have better diets; 2) provide healthier foods at schools; 3) improve access to healthy and affordable foods; and 4) to increase physical activity [71]. To initiate this program, President Obama asked and received from the Congress $1 billion dollars [40].

### Funding for action – infrastructure and programs

Under Obama, a phalanx of new legislation and funding followed suit. Under the 111^th^ Congress (January 3, 2009 – January 3, 2011), Obama and the Congress approved the *Children’s Health Insurance Program Reauthorization*, which provided $25 million for fiscal years 2009-2013 for *Childhood Obesity Demonstration Projects* in communities [70]. The *FIT Kids Act* was also passed, which amended the 1965 *Elementary and Secondary Education Act* in order to improve physical education standards in schools [70]. In December 2010, Obama also penned the *Healthy, Hunger-Free Kids Act,* which increased the number of children eligible for school lunches while mandating an improvement in the nutritional quality of foods provided in schools [72]. That same year Obama also implemented the *Healthy Food Financing Initiative*, a joint project with the US Department of Health & Human Resources, US Department of Agriculture, and the Treasury Department that provided $285 million to help bring more affordable healthy foods to poor urban communities [72]. And in 2011, Obama worked with the US Centers for Disease Control (CDC) to create the *Coordinated Chronic Disease Prevention and Health Promotion Program*, which awards grants to all 50 state health departments to build capacity for addressing chronic illnesses, including obesity, diabetes, cancer, and heart disease [72].

Furthermore, the Affordable Care Act (ACA), which was signed into legislation in 2010, provides $500 million for prevention and wellness grants, increasing to $15 billion in the next 10 years. Moreover, the ACA establishes a *Prevention and Public Health Fund*, providing $12.5 billion in mandatory appropriations for disease prevention, including obesity, over the next 10 years [72]; *Community Transformation Grants*, which allows states to bid for funding to provide safer places for physical activity; a *National Prevention Strategy*, which establishes policies to prevent obesity and its related illnesses, such as diabetes; the *Essential Benefits and Coverage of Preventive Services*, requiring group health insurance plans to cover preventative obesity counseling and annual wellness visits; and the *Children’s Health Insurance Program, Childhood Obesity Demonstration Project*, which, from 2010 to 2014, provides $25 million for comprehensive approaches to reducing obesity among children [72].

Efforts have also been made to help build a safer, more productive physical environment to help reduce childhood obesity. In 2001, the *Carol M. White Physical Education Program* was created in order to provide conditional grants to state health departments in order to purchase equipment and infrastructure for physical fitness in schools, such as slides, jungle gyms, kick balls, and to keep physical education teachers abreast of the latest advances in nutrition and fitness [73]. Since its inception, the program has provided more than $620 million in assistance [73]. In 2012, the US Department of Education, through this program, awarded 56 grants totaling $27 million dollars; and 77 grants exceeding $36.8 million in 2011 [74]. In addition, since 2001 the USDA has provided *Team Nutrition Training Grants*, which although mainly focused on providing healthier meals has also been used for physical education infrastructure [75].

In July 2012, First Lady Obama created the *Let’s Move! Cities, Towns, and Counties initiative*, which builds on the aforementioned 2010 *Let’s Move!* program. This initiative brought together mayors, families, and non-governmental agencies, such as the Blue Cross and Blue Shield Association, to fund the “Play Streets” initiative; this initiative also provides funding to close streets and to provide safer places for families to exercise [76].

Despite these policy efforts, there are limitations, both in the absence and inconsistency of funding to high obesity prevalence areas. For example, although 40 % of the residents in Holmes County Mississippi are obese, they did not receive any federal grant assistance [77]. In 2011, *US News & World Report* also ranked the city of McAllen-Edinburg-Mission, Texas, as the most obese city in America – with an obesity rate of 38.8 % [78]. While McAllen-Edinburg-Mission did received *Carol White* funding for the McAllen Independent School District in the amount of $715,495 in 2011, it did not receive any funding in 2010 or 2009 [78]. The second most obese city, Binghamton, New York (37.6 % obese), did not receive funding for 2011, 2010, or 2009, nor did the third most obese cities, namely Huntington-Ashland, West Virginia, and Ohio County, Kentucky (both 36 % obese) [78].

Second, while the aforementioned federal grants, e.g., *Community Transformation* and *Carol White*, are conditionally based on specific performance standards, annual demonstration of meeting policy goals, and the availability of future local funds [79, 80], they appear not to entail credible disciplinary threats and, thus, incentives for policy compliance. Indeed, it is important to note that these conditionalities have not threatened to withdraw funding for non-compliance to federal guidelines. Rather, guidelines and regulations are heavily *recommended* without enforcement and penalties for non-compliance. Good examples include the *Child Nutrition* and the *WIC Re-authorization Act of 2004*, which required the creation of wellness committees and nutrition standards for all school foods, nutrition education, and physical education [16]. Scholars nevertheless claim that the US Department of Agriculture (USDA) never followed up to ensure that these conditionalities were implemented and if there was an impact on children’s health [16]. Under the *Healthy, Hungry-Free Kids Act*, schools receiving funding for these programs must adhere to new regulations for improved nutritional quality and the provision of water where meals are served [81]. But these regulatory conditions are new and the USDA does not explicitly state what the ramifications are for those state governments failing to adhere to these regulations.

Implemented as part of Obama’s *Healthy, Hungry-Free Kids Act* in December 2010, in 2012 the USDA also announced a farm-to-school program. Through annual disbursements of $5 million dollars, the USDA will provide grants and technical assistance for schools to increase the availability of healthier foods from local farmers; create cooking curricula; build school gardens; encourage field trips to farm producers; and in some instances, establish after school agricultural clubs [81, 82]. Administered through a competitive bidding process, in November 2011, 3000 schools from 27 states were authorized funding [82]. Of the $5 million provided in 2011, $3.5 million was provided in the form of grants, while $1.5 million was provided for technical support and administrative costs [81]. Of these grants, there are two types: planning grants, which provide support for schools that are just starting up farm-to-school initiatives; and implementation grants, for schools that already have an initiative in place [83]. 25 % of all grants were allocated for planning, while the remaining 75 % were allocated for implementation [83].

Through the farm-to-school initiative, the USDA hopes to accomplish several objectives. First it hopes that through field trips to farms, meeting with farmers, and attending cooking classes, children will learn how to eat healthier [83]. And second, in order to ensure that more farmers can participate in these endeavors and provide nutritious foods, the USDA will help farmers acquire GAP (Good Agricultural Producer) certification, which until now has essentially been available only for larger producers that can afford the certification process [83].

Considering the urgent need to help prevent the rise of childhood obesity through better nutrition, as well as the flagging economic performance of small farming businesses, the farm-to-school program is certainly a step forward in the right direction.^3^ But several worrisome issues remain. First, obtaining a farm-to-school grant is a highly competitive process, and with scarce funding available (the program was initially projected to borrow money from USDA programs, such as SNAP [Supplemental Nutrition Assistance Programs]) [84], competition may be fierce. This will inevitably provide advantages for those schools with experienced grant writers and a greater abundance of time and money available to submit strong applications. As mentioned earlier, however, childhood obesity is highly prevalent in the poorest school districts. This leads to the second concern: that the cities with the highest levels of obesity are not receiving farm-to-school funding. Places such as Mississippi’s Holmes County; McAllen-Edinburg-Mission, Texas; Binghamton, New York; Huntington-Ashland, West Virginia; and Ohio County Kentucky will likely receive little, if any, funding.

## Brazil’s response

When compared to the US, Brazil’s response to obesity began at an earlier point in time. By 1996, the Congress had already begun to hold meetings on nutrition education and health, organizing *No Cogresso Nacional de Nutrição* (The National Congress of Nutrition) [48]. Three years later in 1999, after negotiations with the private sector, civil societal actors (such as professors, scientific associations, and workers’ unions), and other federal agencies [15, 85], the ministry of health created the *Política Nacional de Alimentação* - PNAN (National Policy of Nutrition). PNAN officials were cognizant of the obesity epidemic and were working hard to address it through recommendations for enhanced nutrition and the provision of healthier foods [86]. PNAN’s main responsibilities included inter-sectoral cooperation for the universal provision of food; the guarantee of high quality food; the monitoring and reporting of nutritional and food data; the promotion of healthy eating and lifestyles; research; and strengthening human resource capacity in the area of nutrition [86]. Furthermore, PNAN provided discretionary grants to municipal health departments and schools to promote better nutrition and to increase physical activities, as discussed shortly [86]. PNAN also required that 70 % of foods provided for the national feeding program (which included schools) include fresh or minimally processed foods [4, 24]. To that end, PNAN provided grants to farmers for the production of fresh fruits and vegetables for schools and local markets [15].

In addition, in 2000 the congress passed legislation mandating that all packaged foods list their nutritional content, such as calories, protein, carbohydrates, and total fats [24]. This information was provided along with recommended serving sizes and the percentage of daily allowances [24]. It is important to note, however, that this had already been accomplished in the US, beginning in 1990 through the passage of the Nutritional Labeling and Education Act. Nevertheless, Brazil was one of the first nations in the world to legally require the printing of “nutritionally adequate serving sizes” [24], the product of several meetings between the ministry of health and the private sector [24].

Amidst the presence of several innovative policy responses to obesity at the sub-national level [87], the national government escalated its response by 2010. That year, the Congress passed the *Plano de Ações Estratégicas para o Enfrentamento das Doenças Crônicas Não Transmissíveis no Brasil* (DNCT) [88]. This policy established guidelines and procedures for the implementation of anti-obesity initiatives for the next 10 years. In addition to increasing funding for prevention, such as the dissemination of pamphlets, advertisements, and videos in schools (an initiative that began under PNAN), the DNCT also emphasizes training for an effective healthcare staff at the local level [88]. DNCT also includes funding initiatives called the *National Policy and Health Promotion, Physical Activity, & Nutrition* and funding for primary healthcare [88].

In response to the heightened prevalence of childhood obesity in schools, the *Programa Saúde nas Escolas* (PSE) was also implemented via presidential decree No.6.286 in December 2007. PSE is a joint effort between the ministry of health and ministry of education, with the individual – though tightly coordinated – tasks of monitoring and implementing the PSE program [89]. PSE has several priority areas, such as evaluating children’s health conditions; promoting health and prevention; increasing the professionalization of health professionals working on children’s health (with the assistance of the department of education); monitoring and evaluation of student health and programs; increasing health awareness among children, and youth and civil societal participation in policy implementation [90]. Furthermore, the PSE is implemented in two phases: the first entails monitoring and evaluating children’s nutrition, with a careful eye to overweight, obesity, and diabetes; and the second entails providing training for healthcare workers and infrastructural support [89].

Additional legislation was implemented to help ensure that students receive healthier foods in schools. In 2009, law number 11.947 created the *Programa Dinheiro Direto na Escola* (Program for Direct Money to Schools), which provides monetary transfers from the *Fundo Nacional de Desenvolvimento da Educação* (National Development Fund for Education), under the condition that 30 % of the fund transfers purchase foods from local farmers. In abiding by the government’s universal commitment to equality in nutrition and healthcare, funding from the *Programa Dinheiro Direto na Escola* is provided to all communities in need [15].

In 2011, the ministry of health’s *Secretaria de Atenção a Saúde* (Secretary of Attention to Health) also undertook efforts to create an inter-departmental effort to address childhood obesity. The *Secretaria*, through its *Coordenação General de Alimentação e Nutrição* (SAS) – which falls under the *Secretaria’s* Department of Basic Care unit – has been working with officials in the Ministry of Education, Social Development, and Sports to implement new policies. The *Coordenação General* has also adopted the *Programa Saúde nas Escolas* policies and works with the ministry of education, and other federal and state agencies to implement these policies.

And finally, beginning with the *Política Nacional de Alimentação* and now the *Programa Saúde nas Escolas*, the ministry of health also provides grant assistance to purchase physical education equipment. Those states with the most obesity cases have been targeted and provided grants [14]. For example, the municipality with the highest incidence of adult and childhood obesity, with over 21 % of the entire population being obese, Macapá (the capital of the state of Amapá), received assistance; so did the second most obese municipality, Porto Alegre (capital of Rio Grande do Sul), with 19.6 % obese, and the third most obese municipality, Natal (capital of Rio Grande de Norte) [14]. Other states with a high level of obesity, such as São Paulo and Rio de Janeiro, have also received support [87].

While recent grants provided by the *Programa Saúde nas Escolas* are not highly competitive, there are conditionalities imposed. In addition to demonstrating need, state and municipal school boards must agree to comply with the *Programa Saúde nas Escolas’* policy guidelines and expectations [14, 87]. To ensure this, grant money is spread out over several phases [14, 89]. Family Health Program (FHP) teams, who are funded by the ministry of health and that implement *Programa Saúde nas Escolas* policies, repeatedly monitor school district performance in using *Programa Saúde* funding; this, in turn, helps to increase accountability to the latter and the ministry of health. Moreover, any school districts that have agreed to receive money must also organize and participate in the *Semana de Mobilização Saúde no Escola* (Week of School Health Mobilization), which helps to further increase interest and civic participation in preventing childhood obesity and other illnesses [14].

Based on the ongoing success of these funding efforts, in 2011 the ministry of health also created the *Academia da Saúde* (Academy Health Program). Through this program, the ministry provides grant support to over 4000 municipalities to construct parks and to provide free physical education programs [30]. Furthermore, the *Academia da Saúde* provides funding for the hiring of local healthcare personnel that supervise these community fitness programs [30]. And in partnership with municipal health departments, the *Academia da Saúde* provides screening and counseling for healthy lifestyles [30].

Compared to the US, Brazil’s ministry of health also provided grants for farm-to-school programs at an earlier point in time. As mentioned above, this began in earnest under the *Política Nacional de Alimentação* (PNAN) in 1999. Under this program, those states that received grant assistance were required to adhere to the program’s mandate that 70 % of all funds be used to purchase fresh fruits and vegetables from local farmers [15]. Local schools were also encouraged to work closely with community-based organizations [15]. While this conditionality posed a considerable challenge for state and municipal education boards, especially with respect to acquiring the staff needed to clean and prepare foods [15], this conditionality helped ministry of health officials achieve their policy objective [15]. The *Programa Saúde nas Escolas* also provides funding for schools to partner with farmers, as well as helping the latter with financial and technical assistance in producing food [15]. As mentioned earlier, 30 % of all transfers received from the *Fundo Nacional de Desenvolvimento da Educação* must go to purchasing products from agricultural farmers and family businesses [15]. In addition, the *Política Nacional de Alimentação* clearly stipulates that *Programa Saúde nas Escolas* assistance is conditional, based on school board adherence to its policies [15].

### Institutional conversion and social health movements

**Fig. 1:**
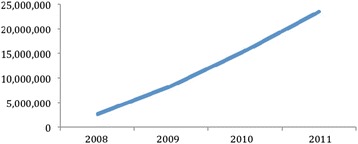
Brazil: Total Number of Family Health Program Teams (2002-2012). Source: Brazil, Ministry of Health, 2012: Data source: http://189.28.128.178/sage/

But what are some of the factors that have led to Brazil’s innovative response to obesity? The design and transformation of historical institutions was important. While national policies and strategies for combating obesity may be new, the institutions used to implement them are not. Building on its rich tradition of centralized intervention for public health [57], in order to ensure that the ministry of health’s policies are effectively implemented it has relied on its preexisting Family Health Program (FHP) and the program’s extensive experience in providing primary care services in rural areas Fig. 1. Created in 1994, the FHP provides primary healthcare (such as physical checkups, drugs, prevention information and referrals) throughout the nation [91]. Funded at a higher pay scale than other physicians employed by state governments, FHP teams are comprised of one primary care physician, one nurse, one nurse assistant, and anywhere between four to six community healthcare workers [91].

**Fig. 2:**
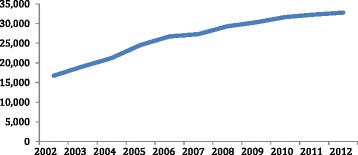
Brazil: Number of Students in Schools where the PSE Program has been Implemented (in millions, 2008-2011). Source: Brazil, Ministry of Health, Program for Health in Schools, 2012

Since the creation of the *Política Nacional de Alimentação* (PNAN) in 1999, realizing the need to strengthen the government’s response to obesity, the ministry of health converted the FHP’s responsibilities to emphasize not only primary care services but also a host of new obesity prevention services [87] Fig. 2. Ministry of health officials realized that rather than dismantling the FHP and/or building new agencies focusing on obesity prevention, they would instead capitalized on the FHP’s long-held tradition of meeting with households and build upon this expertise when assigning FHP teams the task of implementing prevention initiatives, such as helping families craft nutritious meals, monitoring weight, and promoting physical fitness [87]. In recent years, the ministry of health has also relied on the FHP to implement *Programa Saúde nas Escolas* policies [87].

To consolidate and strengthen the FHP’s work, in 2008 the ministry of health, through ordinance No. 154, allocated additional funding to create a FHP support program called the *Núcleo de Apoio á Saúde da Família* (Nucleus of Support for Family Health, NASF) [12, 49]. NASF’s primary function is to supply a team of nutrition specialists, psychologists, physical therapists, educators, and mental health experts that can assist FHP personnel in providing obesity prevention services [12, 49]. The NASF is also expected to provide follow up services with families attended by FHP teams, such as locating healthy foods, proper cooking and nutrition, areas to exercise, and locations for mental health services, while providing more detailed information on specific issues that FHP teams cannot address [12]. By 2011, the ministry of health provided the FHP with 1371 NASF teams in 894 municipalities, signaling a stern commitment to strengthening the FHP’s capacity to assist households [12].

With respect to childhood obesity, because of the limitations in human resource capacity throughout Brazil’s decentralized healthcare system, and because of the difficulty that the public health system has in providing services in hard to reach rural areas, the FHP’s intervention, with the support of NASF, has been critical for ensuring that the *Programa Saúde nas Escolas* work effectively. In addition, FHP personal are instructed to go to schools in areas with the highest levels of childhood obesity, meet with teachers and families, providing them with educational materials about obesity prevention [5, 87, 92].

Throughout the policy reform process, social health movements have also played an important role. Specifically, the historic infiltration of social health movements into the ministry of health, advocating for universal healthcare and nutrition as a human right, inspired essentially all of the aforementioned obesity legislation. Beginning in the 1960s, the *sanitarista* social health movement arose to advocate for healthcare as a human right [59]. Under the military, quality healthcare treatment was relegated only to formal sector employees in the private and public sector [59]. Comprised of medical doctors, healthcare workers, university professors, and politicians, the *sanitarista* movement demanded that the government provide universal healthcare for all, and that it be recognized as a human right [59]. Under the military, the *sanitaristas* successfully infiltrated the highest positions of authority within the ministry of health, gradually building a consensus for reform [59]. Because of their efforts, the 1988 democratic constitution included healthcare as a human right, enshrined through the newly decentralized universal healthcare system [59].

When the challenges of obesity and other weight related disorders emerged, such as type 2 diabetes, these human right principles inspired legislation [48]. These principles were first introduced in 1996, when the government organized the National Conference of Nutrition (*Congresso Nacional de Nutrição*) [48]. During this conference, discussions were held between politicians, health officials, and civil society emphasizing that access to good nutrition is a human right, and that policy should reflect this belief [48]. Policy followed suit: the *Política Nacional de Alimentação* (PNAN) and its focus on increasing awareness of nutrition, funding the provision of higher quality foods through partnerships with farmers was inspired by providing access to nutrition as a human right [93, 94]. The government’s universal distribution of medications for ailments associated with obesity, such as type-2 diabetes, was also deemed a human right issue [93, 95]. Nutrition as a human right also inspired the creation of the *Programa Saúde nas Escolas* program [94].

### But what can be changed in the US?

In contrast to Brazil, in the US there is no history of centralized government intervention and federal programs that the Centers for Disease Control (CDC), which is part of the US Public Health Service, can use to help the states respond to obesity. Rather, public health institutions have historically been fragmented and competitive, incessantly vying for political attention and funding [40]. Furthermore, the US government never created a federal primary healthcare program that would visit families and schools. The closest that the federal government has come to achieving this is the Health Resources and Services Administration (HRSA) community healthcare centers, which provide primary care services to underserved communities and vulnerable populations [96]. However, the HRSA health centers do not provide teams of doctors and nurses visiting households and schools. In fact, it has only been recently proposed that the US Department of Health & Human Services create a group of nurse practitioners that visit households, mainly due to the shortage of doctors and the projected increase in demand for healthcare services in rural areas [10, 11].

Furthermore, in the US a social health movement advocating for access to healthcare as a human right never emerged [60]. Instead, historically social health movements reflected the fragmented and specialized nature of the US public health system: that is, they were focused on access to healthcare for specific diseases, such as polio, malnutrition, and more recently HIV/AIDS [97]. At the same time, the historically powerful influence of the American Medical Association, which built on top of a long tradition of private-based healthcare [60], often successfully pressured presidents and convinced conservative legislators that the idea of access to healthcare as a human right should never emerge [60]. The upshot has been the absence of a social health movement that could emerge within the government and bureaucracy, setting a path for rights-based healthcare and obesity legislation.

## Conclusion

Several important lessons emerge from this comparative case study analysis of the US and Brazil’s response to obesity. First, in order to ensure that obesity prevention policies are successfully implemented, federal health agencies should continue to intervene in local policy implementation by converting preexisting primary healthcare agency subdivisions with extensive experience working with families in order to provide prevention services – as seen with Brazil’s Family Health Program (FHP). Moreover, in order to consolidate this institutional conversion process, federal agencies should create agency subdivisions that support those agencies responsible for providing prevention services, as seen with Brazil’s *Núcleo de Apoio á Saúde da Família’s* (NASF) support for the FHP. Second, the case of Brazil suggests that federal agencies should not only target federal grant assistance to the highest obesity prevalence areas, but that this assistance also entail the usage of aid conditionalities to ensure that funding is used effectively. Finally, findings from Brazil suggests that the bureaucratic infiltration of social health movements advocating for healthcare as a human right is important for inspiring heath officials to create obesity prevention programs, grounded in these normative principles, shaping subsequent legislation.

The case of Brazil also suggests that there is ample room for institutional change theorists and public health professionals to work together in order to understand how decentralized federations can simultaneously encourage the decentralization of health policy while nevertheless creating innovative ways to sustain the federal bureaucracy’s centralized policy influence. The application of institutional conversion theory to federal agencies reveals that governments can sustain their centralized influence by not only converting preexisting agencies for new policy objectives, but also by creating new agencies that reinforce these conversion processes – an issue that the institutional change literature has yet to address [17, 18]. This makes federal agencies working on obesity policy all the more adaptable and capable of intervening at the sub-national level. Centralized institutional conversion processes can also reinforce decentralization processes by ensuring that there is an adequate amount of human resources, experience, and commitment to implementing obesity policies at the local level. Future researchers should therefore strive to combine institutional change theories with an analysis of decentralization and intergovernmental relationships in order to better understand and explain if other nations are capable of approaching Brazil’s institutional approach to obesity prevention.

In the US, despite the introduction of several innovative national obesity awareness campaigns, prevention strategies, and grant programs, none of these institutional innovations were pursued. Nevertheless, considering the similar geographic, infrastructural, and obesity challenges that the US shares with Brazil, US health officials may stand to gain from learning from Brazil’s institutional response to obesity.

For example, building on recent recommendations for providing physician primary care home visits in rural areas [10, 11], the Department of Health & Human Services (HHS) and the Centers for Disease Control (CDC) could create primary healthcare units that proactively work with households and schools to provide obesity prevention services. To create such a program, the Congress and HHS would need to follow Brazil’s lead in providing higher salaries and other benefits to attract these healthcare workers. While a US federal program does exist that provides debt relief and scholarships for primary care doctors agreeing to serve in underserved communities, i.e., the National Health Service Corps, this program is mainly focused on staffing community health centers [98]. Moreover, the National Health Service Corps does not provide regular home visits. Nevertheless, an alternative to creating a new federal program would be to convert the National Health Service Corps’ responsibilities by requiring that staff start providing household visitations for obesity prevention services, especially in distant, poor rural areas.

In addition, the HHS could also follow Brazil’s lead in making sure that those communities with the highest levels of obesity prevalence receive federal grant assistance for physical fitness infrastructure, nutritional, and farm-to-school programs, while facilitating the grant application process. Rather than making grants more competitive, the HHS could focus on imposing stiffer conditionalities and using health officials to carefully monitor the usage of funding, as seen with Brazil’s FHP. This could help to increase local government accountability to the HHS, while possibly enlisting the support of civil societal actors in monitoring grant funding.

But can the US achieve Brazil’s accomplishments in the absence of a long history of federal intervention in sub-national policy? This is possible, for two reasons. First, in periods of healthcare crisis, the government has shown the ability to reform health institutions and policies. For example, under Obama, in response to astronomical healthcare costs, an economic recession, and worsening health indicators, through stern political leadership, democratic party unity and policy compromise, Obama was able to pass the Affordable Care Act (ACA) [99]. The ACA was not only successful in amending the law to increase individual healthcare coverage, but it also entailed a host of new federal programs, spending, and federal regulatory efforts to monitor state insurance programs [99]. Importantly, incremental institutional change occurred despite a long history of successful partisan and private sector resistance to reforming the health insurance system, as well as the absence of historical institutional precedents intervening in state health insurance policy [99]. Second, amidst this critical juncture in US healthcare policy, there is now strong interest among senior health officials to learn from Brazil in order to strengthen the government’s response to obesity [25].

Finally, future researchers may wish to consider how differences in historical, cultural, and social context account for the US and Brazil’s different institutional responses to obesity. For example, when compared to the US, Brazil has a longer tradition of local communities believing that they should always strive to work together in addressing community health issues, family needs, while holding local governments accountable for policy implementation [40]; these ongoing community beliefs, reinforced through historic social health movements believing in the same, such as the *sanitaristas*, may help to account for the Family Health Program’s (FHP) success in working with families. However, these factors were never present in the US, save for brief historic episodes where communities worked together to confront particular diseases, such as smallpox and polio [100]. Brazil’s government also has a longer tradition of emphasizing the practice of home cooked meals [23], which could account for the FHP’s success in working with families to prepare nutritious meals. Given these community and cultural differences, future researchers may wish to consider combining my proposed institutional approach to obesity policy with these broader contextual factors when striving to account for differences in government response to obesity in the US and Brazil.

## Abbreviations

PSF: *Programa Saúde da Familia*
HHS: Health & Human Services
NAFA: National Association for Fat Acceptance
NHANES: National Health & Nutrition Examination Surveys
SNAP: Supplemental Nutrition Assistance Programs
PNAN: *Política Nacional de Alimentação*
DNCT: *Plano de Ações Estratégicas para o Enfrentamento das Doenças Crônicas Não Transmissíveis no Brasil*
PSE: *Programa Saúde nas Escolas*
HRSA: Health Resources and Services Administration
PHS: US Public Health Service
NIH: National Institutes of Health
USDA: US Department of Agriculture
CDC: Centers for Disease Control
NASF: *Núcleo de Apoio á Saúde da Família*
ACA: Affordable Care Act

## Footnotes

1 This study focuses on particular obesity prevention policies, such as those affecting healthy lifestyles and the environment through the government’s funding of parks, gyms, and school playgrounds; the government’s provision of healthy lifestyle and nutritional information to families and schools; and the government’s provision of nutritious foods in schools through mandated school partnerships with farmers.

2 Henceforth the terms “reform actor” or “change actor” will refer to those individuals within institutions seeking to transform it in order to achieve their objectives.

3 There is no guarantee and evidence that the presence of farm-to-school programs reduces childhood obesity. Nevertheless, recent studies suggest that these programs have improved children’s eating habits, such as consuming more vegetables and fruits; increased children’s knowledge of healthier food options; and increased their participation in healthier school lunch programs [101].
